# ‘Telling our story... Creating our own history’: caregivers’ reasons for participating in an Australian longitudinal study of Indigenous children

**DOI:** 10.1186/s12939-018-0858-1

**Published:** 2018-09-15

**Authors:** Katherine Ann Thurber, Anna Olsen, Jill Guthrie, Rachael McCormick, Andrew Hunter, Roxanne Jones, Bobby Maher, Cathy Banwell, Rochelle Jones, Bianca Calabria, Raymond Lovett

**Affiliations:** 1 National Centre for Epidemiology and Population Health, Research School of Population Health, The Australian National University, Acton, ACT 2601 Australia; 2 Medical School, The Australian National University, Acton, ACT 2601 Australia

**Keywords:** Indigenous population, Longitudinal studies, Research design, Trust, Ethics, Motivation

## Abstract

**Background:**

Improving the wellbeing of Indigenous populations is an international priority. Robust research conducted with Aboriginal and Torres Strait Islander peoples is key to developing programs and policies to improve health and wellbeing. This paper aims to quantify the extent of participation in a national longitudinal study of Aboriginal and Torres Strait Islander (Indigenous Australian) children, and to understand the reasons why caregivers participate in the study.

**Methods:**

This mixed methods study uses data from Wave 6 of *Footprints in Time*, the Longitudinal Study of Indigenous Children. We conducted descriptive analysis of quantitative variables to characterise the sample and retention rates. We applied conventional content analysis to 160 caregivers’ open-ended responses to the question, ‘Why do you stay in the study?’, identifying themes and overarching meta-themes.

**Results:**

The study has maintained a high retention rate, with 70.4% (*n* = 1239/1671) of the baseline sample participating in the study’s 6th wave. We identified seven themes related to why participants stay in the study: telling our story, community benefit, satisfaction, tracking Study Child’s progress, study processes, receiving study gifts, and valuing what the study stands for. These related to two meta-themes: reciprocity, and trust and connection. Caregivers reported that participation was associated with benefits for their family and community as well as for the study. They identified specific features of the *Footprints in Time* study design that built and maintained trust and connection between participants and the study.

**Conclusions:**

Our findings support the assertion that Aboriginal and Torres Strait Islander people want to be involved in research when it is done ‘the right way’. *Footprints in Time* has successfully recruited and retained the current-largest cohort of Aboriginal and Torres Strait Islander children in Australia through the use of participatory research methodologies, suggesting effective study implementation and processes. Participants indicated ongoing commitment to the study resulting from perceptions of reciprocity and development of trust in the study. *Footprints in Time* can serve as a successful model of Aboriginal and Torres Strait Islander health research, to promote good research practice and provides lessons for research with other Indigenous populations.

## Background

Improving the wellbeing of Indigenous populations is an international priority [1]. Aboriginal and Torres Strait Islander peoples are Australia’s First Peoples, and maintain some of the longest continuing cultures in the world [2]. The 3% of Australians who identify as Aboriginal and/or Torres Strait Islander are a heterogeneous peoples, comprising a diversity of cultures and experiences. The intergenerational impacts of colonisation include trauma, forced disconnection from land and culture, persisting socioeconomic disadvantage, and systemic, institutional, and interpersonal racism. While Aboriginal and Torres Strait Islander peoples have demonstrated resilience in the face of this, the population is over-represented in poor health and wellbeing outcomes [3, 4]. Many of the health and social inequities experienced by Aboriginal and Torres Strait Islander peoples in Australia are also experienced by other Indigenous populations internationally [1].

Robust research conducted with Aboriginal and Torres Strait Islander peoples is key to developing programs and policies to improve health and wellbeing [1, 5, 6]. Holistic views of health are commonly held by Aboriginal and Torres Strait Islander peoples, encompassing a whole-of-life view of the social, emotional, and cultural wellbeing of the community, as well as the individual’s own physical health and wellbeing [7, 8]. To enable meaningful analysis of Aboriginal and Torres Strait Islander wellbeing, it is therefore critical that studies collect comprehensive data on a broad range of social, cultural, and environmental factors at the individual, family, and community levels. Longitudinal studies are particularly valuable as they enable examination of the complex interplay of factors on wellbeing across the life course, including providing insight into causal pathways [9, 10].

It is challenging in any population to retain participants in a longitudinal study [11, 12], which may have implications for the validity of the data and findings. There is some evidence that retention rates are lower for Aboriginal and/or Torres Strait Islander participants compared to non-Indigenous participants in large-scale Australian longitudinal studies [13–16]. For example, retention of Aboriginal participants at the first wave of follow-up in the 45 and Up Study was significantly lower than retention of non-Aboriginal participants (45% versus 61%; age-sex-adjusted participation rate ratio 0.72, 95%CI:0.66,0.78) [16]. Lower retention rates may reflect additional barriers to research participation for Aboriginal and Torres Strait Islander, compared to non-Indigenous, Australians. Many Aboriginal and Torres Strait Islander communities face a large respondent burden, with frequent invitations to participate in research [17, 18]. Aboriginal and Torres Strait Islander peoples are more likely than non-Indigenous people to live in hard-to-reach areas, and to be mobile, which can make following and communicating with participants challenging [9, 18–20]. In addition, many Indigenous populations internationally share a history of negative and exploitive research practices, which has had a lasting legacy, including enduring mistrust in research [9, 21–24].

Despite potential challenges recruiting and retaining participants, some longitudinal studies of Aboriginal and Torres Strait Islander children and adults exist [25–27]. In addition to improving understanding of Aboriginal and Torres Strait Islander peoples’ health and wellbeing, these studies provide an opportunity to better understand enablers of research participation by Aboriginal and Torres Strait Islander peoples. This information can inform development of future studies, to improve retention and the validity of data. It can also support the conduct of ethical and respectful research, enabling positive research experiences and outcomes for Aboriginal and Torres Strait Islander peoples.

Previous literature, from the perspective of researchers, has identified contributors to the retention of Aboriginal and Torres Strait Islander participants in longitudinal studies, including the use of Indigenous research methodologies, partnerships and relationships between researchers and community members, flexibility, transparent communication, and cultural sensitivity [9, 12, 18, 27, 28]. To our knowledge, only one peer-reviewed study has examined reasons for research participation from the perspective of Aboriginal and Torres Strait Islander peoples, and this study was restricted to a small (*n* = 8), localised sample [5]. The current analysis serves to extend this knowledge by exploring perspectives on research participation in a larger, heterogeneous sample. The aims of this mixed methods paper are to quantify the extent of participation in a national longitudinal study of Aboriginal and Torres Strait Islander children, *Footprints in Time,* and to understand (qualitatively) the reasons why caregivers participate in the study.

## Methods

### Study population

This paper uses data from *Footprints in Time*, the Longitudinal Study of Indigenous Children (LSIC), a national study managed by the Australian Government Department of Social Services, and overseen by an Aboriginal-majority Steering Committee. The Steering Committee were key advisors in the development of the *Footprints in Time* study, including ensuring extensive community engagement and a participatory approach [28]. Starting in 2003, representatives from the *Footprints in Time* study held 23 consultation meetings with Aboriginal and Torres Strait Islander stakeholders; meetings were held in every capital city and at least one regional or remote area in each State and Territory [27]. The study then trialled data collection methods and community engagement and dissemination strategies in three geographic areas, from 2004 to 2005 [27]. Based on these consultations, the study’s primary research question is, ‘What do Aboriginal and Torres Strait Islander (Indigenous) children need to have the best start in life to grow up strong?’ [29].

In 2008, Aboriginal and Torres Strait Islander children aged 0.5–2.0 years and 3.5–5.0 years were recruited through purposive sampling. Follow-up surveys are conducted annually, and the study is ongoing. The sample includes a total of 1759 children, representing 5–10% of the total Aboriginal and Torres Strait Islander population in these age groups, and their caregivers. *Footprints in Time* is not intended to be representative of all Aboriginal and Torres Strait Islander families, consistent with its longitudinal study design [30]; it is intended to provide a picture of life in a range of different environments by sampling from 11 diverse sites across Australia [27]. Further details on the study design are provided elsewhere [27].

Aboriginal and Torres Strait Islander Research Administration Officers (RAOs) conduct face-to-face surveys with participating families, usually in the family’s home. RAOs often live in the region in which they conduct interviews, and where possible, the same RAO conducts the survey with each family from one year to the next [18, 31, 32]*.* Separate interviews are conducted with multiple informants within each family including the study child and their primary caregiver [27]. The primary caregiver is usually the mother or step-mother, but can also be the Grandmother, Aunty, father, step-father, or other, reflecting the diversity in structure and composition of Aboriginal and Torres Strait Islander families [33]. In addition to collecting quantitative data, qualitative data are collected through ‘free-text’ responses to open-ended questions [18]. Responses are transcribed verbatim or summarised by RAOs with the assistance of computer technology. These responses are confidentialised prior to their release to remove any potentially identifying information [18].

Interviews with the primary caregiver are the most comprehensive, ranging in time from 20 min to 3 h, and include questions about the study child, their caregivers and other family members, the household, and the community [27]. Interviews with the study child are shorter, ranging in time from two to 50 min [27]. Across waves, the average total household time per survey is around 1.5 h [34].

To support ongoing relationships with participants and communities, *Footprints in Time* has developed a feedback and dissemination strategy, which includes: internal feedback loops to incorporate community and RAO input into survey design; sending Community Feedback Sheets, which provide results specific to each of the 11 sites; Community Booklets, which summarise findings across the cohort; and, Research Feedback Sheets based on specific research projects [27, 35]. The study results and information are provided in accessible, plain language. Families receive incentives for participation at each wave of the study, which have included t-shirts and towels. Every year, participating families also receive a *Footprints in Time* calendar that includes photos of participating children, taken (with consent) at the previous wave.

In this paper we examine data from families who participated in Wave 6 of *Footprints in Time* (collected in 2013), using Data Release 7.0. All data utilised in this paper are self-reported by the primary caregiver in the face-to-face interview, except remoteness and area-level disadvantage, which are derived from participants’ addresses.

### Research methodology

Indigenous ways of knowing and participatory methodology formed an overarching research model for this analysis. Indigenous ways of knowing involves grounding the research in a model that respects cultural history, knowledge, and protocols [36]. Approaches to participatory research (action research, experience-based co-design, participatory action research, community-based participatory action research) involve collaboration between researchers and community [37, 38]. Instead of seeing ‘experts’ (e.g. university researchers) as the only legitimate source of knowledge, participatory research recognises and values the knowledge of community members. Participatory research models are intended to challenge researchers to share influence and control over aspects of a research project such as questions and design, research processes, data collection, interpretation, dissemination, and translation. Participatory research is increasingly popular with Indigenous communities as the approach can counter the colonising effects [39] of historical research on Indigenous peoples, and can help avoid the misrepresentation of ‘Indigenous societies, culture and persons by non-Indigenous academics and professionals’ ([39] p. 855).

Meaningful engagement of community members in research encourages the building of trusting relationships, establishment of new data collection methods, shared interpretation of results, and mutual benefit. Participatory approaches aim to generate research findings that are ‘useful and useable to all of those participating in the process’ ([40] p. 190). To achieve this, people who are members of the community are often engaged as researchers (community researchers, co-researchers, peer researchers) [38].

Both the original study (*Footprints in Time*) and this secondary analysis of quantitative and qualitative data from the study draw on participatory research methodologies. In *Footprints in Time*, this was achieved through processes including ongoing consultation and feedback processes, employment of Aboriginal and Torres Strait Islander RAOs, and involvement of the Aboriginal and Torres Strait Islander-majority Steering Committee (described above) [28]. Following the participatory structure of *Footprints in Time*, the approach and analysis employed in this paper were co-designed by Aboriginal and non-Aboriginal researchers (including one Aboriginal member of the Steering Committee) and *Footprints in Time* community researchers (RAOs). A knowledge exchange focus group was held in July 2017 with eight *Footprints in Time* RAOs to discuss and contextualise preliminary findings, and synthesise key messages to include in a research feedback sheet for participants. The RAOs’ reflections are incorporated into the results and discussion sections.

### Variables

#### Quantitative data

Characteristics of the sample reported in this paper comprise: primary caregiver age, gender, and identification as Aboriginal and/or Torres Strait Islander; the relationship between the study child and their primary caregiver; the level of geographical remoteness (measured according to the Level of Relative Isolation scale); and, the number of waves of *Footprints In Time* in which families participated, up to and including Wave 6.

#### Qualitative data

Qualitative data included in this paper comprise responses from primary caregivers when asked, ‘Why do you stay in the study?’, with the follow-up prompt, ‘What do you like about Footprints in Time?’. Participants could provide a response, indicate that they did not know why they stayed in the study, or choose not to provide any response.

### Analysis

We conducted descriptive analysis of quantitative demographic variables to characterise the sample using Stata 14.

Qualitative data were analysed using conventional content analysis [41] and managed using Microsoft Excel. In the first phase, 130 free text responses (approximately 10% of the sample) were randomly selected for analysis.

The procedure of analysis was informed by existing models [41–43]. Guided by the survey questions along with an inductive approach to establishing themes [41, 42], three analysts (AH, RM, KT) undertook the qualitative data analysis. Each analyst independently read the complete transcript and re-read responses line-by-line before reflecting and identifying preliminary themes. They then systematically coded the text using preliminary themes, aiming to stay close to the text rather than trying to infer underlying meaning(s) [42]. The analysists then met as a group to compare their initial themes and to work towards an agreed theme structure for the initial sample of 130 responses.

Data were coded using the theme structure developed by the group. An additional random sample of 30 responses was reviewed to assess if saturation had been reached [44–47]; no new themes were identified. This analysis includes the initial set of 130 responses and additional 30 responses, for a total of 160 responses (our subsample).

Following Onwuegbuzie’s method for ascertaining frequency effect sizes in qualitative data [48], the next step was to count the number of times a theme was identified (frequency). The next step involved interpretative analysis, comparing and contrasting themes in order to elucidate relationships between themes and to develop meta-themes, providing an overarching framework to interpret findings. A focus group was then held with *Footprints in Time* RAOs to check the theme structure and to contextualise themes and meta-themes.

## Results

### Retention rate and sample characteristics

**Fig. 1:**
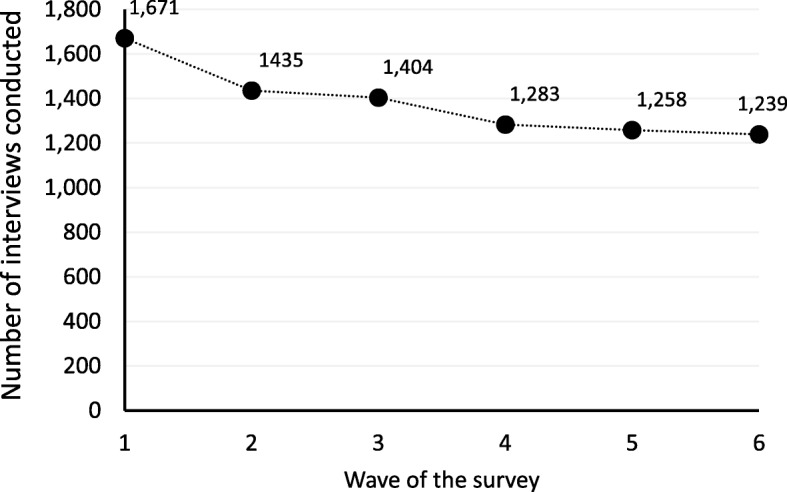
Number of participating families in Waves 1 to 6 of *Footprints in Time*. * Numbers refer to interviews with the primary caregiver. The total sample of 1759 families includes 1671 Wave 1 (baseline) participants and 88 new entrants who joined the study in Wave 2

**Fig. 2:**
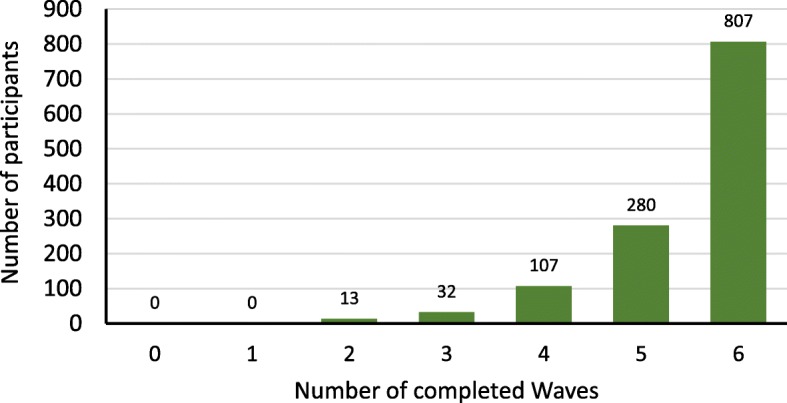
Number of *Footprints in Time* interviews completed by Wave 6 participants. * Numbers refer to interviews with the primary caregiver. This graph presents the number of LSIC interviews completed by families participating in Wave 6 of the study. The minimum number of completed interviews is two interviews, as families needed to have participated in the first or second wave to be part of the cohort, and needed to have participated in Wave 6 to be included in this analysis. The maximum number of completed interviews is six interviews, representing families who participated in every single wave of the survey up to that date

The families participating in Wave 6 of *Footprints in Time* represented 70.4% (1239/1671) of the baseline sample (Fig. 1). Sixty-five percent (*n* = 807/1239) of families participating in Wave 6 had participated in all six waves of the study to date (Fig. 2), corresponding to six years of involvement in the study.

Over 90% (*n* = 1133/1239) of caregivers participating in Wave 6 provided a free-text response explaining why they chose to participate in *Footprints in Time*; the remaining caregivers either did not provide a response or responded that they did not know.

Characteristics of caregivers in our sub-sample and of all families participating in Wave 6 are presented in Table 1. Characteristics of our sub-sample were similar to those of the total sample. The majority of caregivers identified as Aboriginal and/or Torres Strait Islander, were female, and were the study child’s mother or step-mother; a small proportion were fathers or other relatives. Given the small number of male primary caregivers in our sample, the gender of caregivers will not be linked to their responses in order to protect confidentiality. A high proportion of participating families lived in inner regional areas, followed by major cities, remote/very remote settings, and outer regional areas.

**Table 1:** Profile of caregivers in the content analysis sub-sample (*n* = 160) and the total Wave 6 sample (*n* = 1239)

	Distribution in content analysis sub-sample		Distribution in full sample	
	%	(n/N)	%	(n/N)
PRIMARY CAREGIVER CHARACTERISTICS				
Gender				
Female	96.3	(154/160)	96.9	(1201/1239)
Male	3.8	(6/160)	3.1	(38/1239)
Age (years)				
21–30	33.1	(53/160)	29.7	(368/1239)
31–40	45.0	(72/160)	46.3	(574/1239)
41 and over	21.9	(35/160)	24.0	(297/1239)
Indigenous identification				
Aboriginal	69.4	(111/160)	71.0	(879/1238)
Torres Strait Islander	8.8	(14/160)	7.4	(92/1238)
Aboriginal and Torres Strait Islander	5.0	(8/160)	3.7	(46/1238)
Neither Aboriginal nor Torres Strait Islander	16.9	(27/160)	17.9	(221/1238)
Relationship to study child				
Mother or step-mother	88.8	(142/160)	88.0	(1090/1239)
Father or step-father	3.8	(6/160)	2.9	(36/1239)
Grandmother, Aunty, or other	7.5	(12/160)	9.1	(113/1239)
HOUSEHOLD CHARACTERISTICS				
Level of remoteness				
Major city	24.4	(39/160)	27.8	(344/1239)
Inner regional area	51.3	(82/160)	50.8	(629/1239)
Outer regional area	10.6	(17/160)	12.8	(158/1239)
Remote or very remote	13.8	(22/160)	8.7	(108/1239)
Number of Waves of LSIC completed				
2–4	11.3	(18/160)	12.3	(152/1239)
5	24.4	(39/160)	22.6	(280/1239)
6	64.4	(103/160)	65.1	(807/1239)

*Numbers may not sum to total due to missing data. Level of remoteness defined according to Level of Relative Isolation

### Thematic analysis

Seven themes related to why participants stay in the study were identified in the content analysis: telling our story, community benefit, satisfaction, tracking Study Child’s progress, study processes, receiving study gifts, and valuing what the study stands for. Theme definitions and frequency of their occurrence are provided in Table 2.

**Table 2:** Themes, definition and frequency

Theme	Sub-themes	Frequency (% of total)	Definition	Example quote	Meta-theme	
					Reciprocity	Connection and trust
Telling our story	Contributing information; data about Indigenous kids; educating the public; recording what life is like; protecting and maintaining culture.	42 (26.3%)	Refers to recording and contributing information, including for research, government, organisations, and the public.	‘Telling our story... creating our own history’	X	
Community benefit	–	33 (20.6%)	Refers to perceived benefit for the broader Aboriginal and Torres Strait Islander community and future generations.	‘I think it is deadly this sort of research, it will all come together and help our kids in the future’	X	
Satisfaction	P1 enjoys or finds it interesting; SC enjoys; good for SC; connect SC to Aboriginality and culture.	37 (23.1%)	Refers to caregivers or children’s satisfaction through participating in the study, such as enjoyment and interest.	‘I really like it. I love it!’	X	
Tracking Study Child’s progress	Track progress and set goals; a record or time capsule; independent way to monitor SC; help P1 to understand SC.	35 (21.9%)	Refers to recording or tracking how their child is progressing over time.	‘Am able to see how my son is improving and progressing each year’	X	
Study processes	Time; feedback sheets; building relationships; confidentiality.	36 (22.5%)	Refers to specific study processes, such as the timing of interviews and feedback processes.	‘The feedback is really helpful’	X	X
Receiving study gifts	–	55 (34.4%)	Refers to gifts or incentives provided to participants by the study.	‘… enjoy seeing the excitement on my child’s face when they have been given gifts’	X	
Valuing what the study stands for	Support the study; focus of the survey on Aboriginal children, culture.	14 (8.8%)	Refers to perceived value of the study, including the importance of the study’s focus and its findings.	‘… because I like what it stands for’		X

*SC* Study Child*, P1* Primary caregiver

**Fig. 3:**
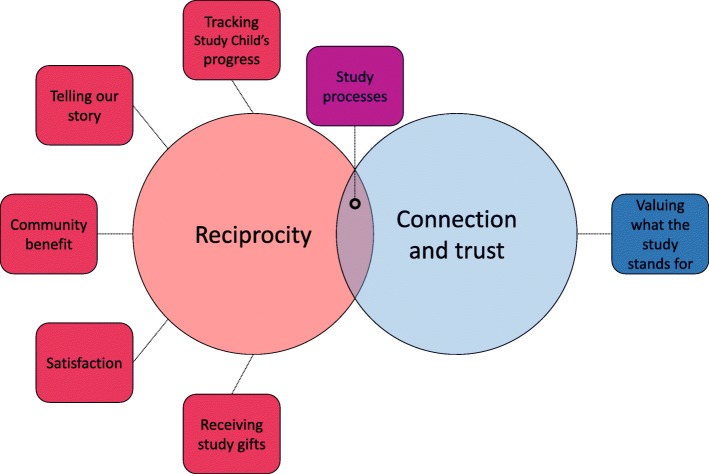
Relationships between themes and meta-themes

Noting conceptual interrelationships between the themes, our final analytical step involved exploring the underlying meanings within our theme structure, from which we developed two overarching categories: (1) reciprocity and (2) trust and connection. We use these meta-themes and their interrelationship to describe findings below (see Fig. 3).

### Meta-theme: Reciprocity

Research participation is often presumed to be altruistic (i.e. participants provide their time, their body, or their information for science or public good). However, caregivers in this study reported that, in addition to being beneficial for the study, they felt that their participation was associated with benefits for their family and community. These benefits included the exchange of information (telling our story; tracking Study Child’s progress; study processes), enjoyment and developing relationships with interviewers (satisfaction; study processes), incentives for participation (receiving study gifts), and benefits for the broader community (community benefit). Reciprocity encapsulates this mutual benefit.

#### Telling our story

Over a quarter of responses (26.3%, *n* = 42/160) encompassed the concept of ‘telling our story’. These focused on the value of ‘recording what life is like’; contributing to official statistics, research, government, organisations, or other; preserving and sharing culture; and collecting data about Aboriginal and Torres Strait Islander children.

Many caregivers specifically identified value in ‘keeping data on’, ‘keeping track of’, and ‘keeping an eye on’ all Aboriginal and Torres Strait Islander children as exemplified by statements such as:

*I think it is a good idea to gather information about all the children that live in different environments.*

Caregivers not only described participation as an opportunity to create a repository of information about Aboriginal and Torres Strait Islander life, but also as an empowering experience of recording culture and diversity from their perspective:

*…. telling our story... creating our own history.*

*I want our culture to stay strong and the only way to do that is to record the information that is out there.*

Caregivers felt that the study findings could be used to support education about Aboriginal and Torres Strait Islander life, in particular, positive or ‘good’ stories:

*I think it is a very good idea for this information to get out to the community so that we can educate other people about our people and our culture.*

*I want the general public to know that there are good statistics on Indigenous children.*

Caregivers expressed the desire for information from the study to be ‘fed to organisations and the government.’ Caregivers explained that it was important to provide information to:

*… get the right statistics for government*

*… help the government understand the needs of Aboriginal [people]*

In some cases, respondents anticipated that this community benefit could arise through identifying methods for improvement, informing policy, or attracting funding or resources:

*I hope the Government will listen to what we are saying*

*… hopefully it will change where they direct the funding*

*If you don't know what's broken you don't know how to fix it*

#### Community benefit

More than one in five caregivers (20.6%, *n* = 33/160) mentioned an anticipated benefit for the broader Aboriginal and Torres Strait Islander community and future generations – not just their own child or their own community. Responses focused on making a positive difference in the community through the identification of ‘good things’, ‘ways of improving’, ‘help’ and ‘funding’. Community benefit was not anticipated to be immediate but over the long-term. Many caregivers mentioned that participating in the study would contribute to a better future for Aboriginal and Torres Strait Islander people; for example, ‘Looking forward to the future’; and to ‘see outcomes in the long run’. These future gains were particularly important for their children:

*… the study is useful for the future; our kids are our future.*

*I think it is deadly this sort of research, it will all come together and help our kids in the future.*

#### Satisfaction

Almost one quarter of caregivers (23.1%, *n* = 37/160) described satisfaction from, enjoyment in, and perceived benefits from participating in the study, for both themselves and their child. Caregivers reported that they found the study ‘interesting’, and that they enjoyed participating in the study and being a part of the annual interviews, providing comments such as:

*I like being in the study… I like these interviews.*

*I really like it. I love it!*

Many caregivers reported that their child enjoyed doing the survey (particularly the activities), and that their child looked forward to participating: ‘It is something for the [child] to look forward to’. Caregivers’ responses indicated that they perceived participation in the study was inherently good for their child:

*I like to have positive outcomes for my children.*

*It’s helping [child] along.*

During the focus group, RAOs explained that participating in *Footprints in Time* is an important way for participants to reconnect with culture, particularly for families who feel disconnected. This connection can be fostered through the provision of time and a safe space for participants to answer questions about culture and think about ways to be involved with culture. RAOs reported facilitating cultural connection not only through the study visits, but also by sharing information with families about local cultural groups and events. Echoing the RAOs perspective, several caregivers mentioned that participation in the study strengthened children’s connection to their culture, such as:

*… help [child] to connect with her Aboriginality.*

*Link Aboriginal children with their culture.*

#### Tracking study Child’s progress

Thirty-five caregivers (21.9%) reported that the ability to record and track their own child’s development was a reason for participating in the study. Participating in the study provides families with an opportunity to see how their child is progressing, in terms of growth, development, and schooling:

*[I] am able to see how my son is improving and progressing each year.*

Through participating in the study, families felt that they were better able to understand their child and to set goals for the child:

*…gives parents a better understanding of their children.*

*… it helps me to understand my daughter more as she is growing up.*

*… makes families look at the goals for their Aboriginal children.*

Caregivers mentioned that they valued that the study provided an opportunity for independent monitoring of the child, outside of the immediate family. For example, one caregiver reported that through participating in the study she ‘… would be able to see if she was doing the right thing with her parenting’.

Caregivers also commented on the value of creating a record or ‘time capsule’ of their child. For example, one mentioned that participating allows her to ‘… see the history and see the progress as she gets old. Like the funny things we just put in it will be there forever...’. Others mentioned the value on having the data recorded:

*... have something to look back on*

*Knowing that I can look back at this information in years to come and see where [child] is as a person*

#### Study processes

Many responses referred to specific features of the *Footprints in Time* study design, such as the feedback sheets sent to participating families every year, which represent another form of knowledge exchange and reciprocity. These feedback processes allow families to track progress in their community and in the cohort overall, and to stay informed of research findings from the study. For example, caregivers said:

*… it is good that Footprints keep in touch.*

*I like to read the reports that are sent out - the feedback sheets.*

*It is interesting to see the newsletter and the graphs.*

*The feedback is really helpful.*

Other study processes that were described positively by participants are described below under the meta-theme trust.

#### Receiving study gifts

The incentives provided in *Footprints in Time* were appreciated by participating children and families, and also served to connect participants to the study.

Over a third (34.4%, *n* = 55/160) of caregivers mentioned that they accepted and enjoyed the gifts received for participating in the study; multiple responses mentioned the calendar: ‘always look forward to the calendar’; ‘the kids get to be in the calendar’. Caregivers also reported enjoyment in ‘seeing the excitement on my child’s face when they have been given gifts’.

During the focus group, RAOs explained that the incentives provided also enabled children and families to connect to their Aboriginal and/or Torres Strait Islander identity; for example, gifts with *Footprints in Time* logos reinforce the child’s identification as a participant in the study, along with a sense of pride and connection.

### Meta-theme: Connection and trust

Participants’ responses suggest that specific features of the *Footprints in Time* study design (study processes) worked to build and maintain trust and connection, or rapport, between participants and the study. For example, the longitudinal study design, and continuity of RAOs across study waves, ensures frequent contact and enables relationship building between participants and the study. Participants also reported that they valued ‘what the study stands for’, suggesting that the study topic is of interest and priority to participating families and communities, supporting their trust in and connection to the study.

#### Study processes

Thirty-six caregivers (22.5%) mentioned specific features of *Footprints in Time* that contributed to their participation, and fostered a sense of trust in and connection to the study.

Many responses alluded to the concept of time, particularly that the regular contact with the study was a positive experience. Several responses referred to the consistency of the annual visits, commenting, ‘the service is regular’ and ‘that you came out to visit my child every year’. One commented that she appreciated that the survey ‘doesn’t take much out of your time’ (despite the fact that the average participating household spends 1.5 h on each annual survey).

Responses conveyed the development of trust and rapport between participants and RAOs over the course of the study. Multiple caregivers commented that having the same RAO every year enabled them to build a relationship, with comments including:

*If it was a different person each year I probably wouldn't do it*

*I like [RAO name] doing my survey, I don't like change*

In the focus group the RAOs also reflected on the establishment of relationships with participating families. For example, one RAO explained that over the course of three annual surveys the RAO progressed from conducting the survey outside one family’s house to being welcomed inside their home.

Many caregivers described a strong sense of connection to and trust in the RAOs and appreciated the social support received through engaging with the RAOs during the annual face-to-face interview.

*I love you guys [RAOs]… you are all lovely people*

*… we love you.*

*I feel very comfortable having the RAOs in my home.*

*… the interviewers are friendly and easy to talk to about the questions.*

Some reported that they enjoyed telling the interviewers about their child and ‘having a yarn’ (yarning is an Aboriginal term used to describe talking or telling stories, a process through which knowledge has been transmitted across generations [7], and through which connections and relationships can develop [49]). Several stated that the study interviews were not intrusive, ‘respectful of the community’, and the ‘best way to research’. One participant explicitly stated that she appreciated that the interviews were conducted by Aboriginal and Torres Strait Islander, rather than non-Indigenous, people. During the focus group RAOs explained that they follow community protocols and reschedule interviews if families are undergoing difficult personal circumstances or if there is an event occurring in the community.

#### Valuing what the study stands for

Fourteen caregivers (8.8%) reported that they were involved in *Footprints in Time* because of ‘what it stands for’. Continuing the theme of connection, participants appreciated that the study asks questions about things of value to Aboriginal and Torres Strait Islander families, allowing them to share parts of their life and ‘story’ that are important to them. Participants valued the holistic approach to considering the broader family and community context beyond the individual child, as well as the centrality of culture to the study. For example, one participant commented that she appreciated that the study wanted ‘to know about our culture and what [place] is like’.

The most common value that connected participants to the study was its focus on children, and in particular, the focus on improving Aboriginal and Torres Strait Islander children’s wellbeing:

*It's good that the study takes an interest in our children's development.*

*I believe that it is good to focus on the needs of Aboriginal children.*

*… there is an organisation out there that has time to care about our kids.*

## Discussion

*Footprints in Time* has successfully recruited and retained the current-largest cohort of Aboriginal and Torres Strait Islander children in Australia. More than 8000 surveys were completed across the first six waves of the study, from 2008 to 2013; this represents a contribution of around 12,000 h of time by families of Aboriginal and Torres Strait Islander children. At the 6th annual survey, the study had retained over 70% of the total cohort; the majority (65%, *n* = 807/1239) of families who participated in Wave 6 had participated in every one of the preceding five surveys. The qualitative data collected in *Footprints in Time* provides valuable insights as to why the study was successful in retaining families. Namely, participants indicated ongoing commitment to and interest in participating in the study due to perceptions of reciprocity and the development of trust in the study. This was enabled by the participatory approach to developing and implementing the study.

There is no established definition of a satisfactory retention rate, but previous studies of Aboriginal and Torres Strait Islander peoples have described retention rates between 45 and 85% as satisfactory [12, 16, 19, 25]. The retention achieved in *Footprints in Time* (70.4% at Wave 6) matches that of longitudinal studies of the total Australian population conducted by Department of Social Services. The Longitudinal Study of Australian children maintained 72.4% of the baseline sample at the 6th wave of follow-up (*n* = 7301/10,090); the study of Household, Income and Labour Dynamics in Australia maintained 72.2% of the baseline sample at the 6th wave of follow up (*n* = 12,905/13,969) [50]. The ability of *Footprints in Time* to maintain an equivalent response rate despite additional complexities (including high mobility and respondent burden, and negative research experiences [12, 16, 19, 51, 52]) suggests effective study methodology and implementation.

The two meta-themes identified in this study, reciprocity and trust/connection, align with key ethical principles for the conduct of research with Aboriginal and Torres Strait Islander peoples [53–55] (and other Indigenous populations internationally, e.g. [56, 57]), reinforcing the importance and appropriateness of these principles. For example, reciprocity is a core principle for the conduct of ethical Aboriginal and Torres Strait Islander health research, recognising that research participants – not just the researchers – need to gain from the research process. This contrasts common experiences of Aboriginal and Torres Strait Islander research that has ‘taken away’ but not ‘given back’ to the community [58–60].

Reciprocity encompasses two key components: benefit and inclusion [52, 55]. The first component, benefit, entails the enhancement of capacities, opportunities, or outcomes of interest and value to Aboriginal and Torres Strait Islander peoples [55]. Respondents described a number of valued individual- or family-level benefits to participating [61]; this included the identity affirming nature of the study, study incentives, mutual knowledge exchange [22, 58, 59], and satisfaction inherent to completing the annual surveys. Responses also described anticipated benefits for the broader community over the long term. The emphasis on community, in addition to individual, benefit is consistent with previous research [5] and with holistic and collective views of wellbeing often held by Indigenous peoples [7, 8]. Respondents’ views also indicate that they felt included and valued as members of the study, particularly in relation to knowledge exchange. Inclusion in research entails ‘equitable and respectful engagement with Aboriginal and Torres Strait Islander Peoples, their values and cultures in the proposed research’ ([55] p. 10). This second component of reciprocity is tightly linked to trust, and is facilitated by the study’s ongoing community engagement and feedback processes, and by RAOs’ flexibility and respect for families and community protocols. Establishing study designs in which information is provided to participants, not just taken, is considered an essential component of collaborative, decolonising methodologies [22, 58, 59].

Connection, particularly as a facilitator of trust, has similarly been identified as a key element of ethical research practice [5, 62]. Australia’s key guidelines for ethical research with Aboriginal and Torres Strait Islander communities include principles related to generating and maintaining trust and integrity in research [53–55], which can be supported through reciprocity [62]. Participants contribute their time, body, and/or information to research, trusting the researchers that this will at some point translate to benefit [62]. As described under reciprocity, our findings suggest that caregivers trust that their participation in the *Footprints in Time* study will generate valued benefits for their family, and for the broader community over the long term. Participation in research also requires trust that any data collected will used appropriately. Data about Aboriginal and Torres Strait Islander people have often been misused and have misrepresented participants; literature has documented ongoing concerns about the use of data [5, 58, 59]. Several participants expressed their confidence that the study would generate ‘good’ or the ‘right’ (not just any) statistics about Aboriginal and Torres Strait Islander children and families. That is, participants placed trust in *Footprints in Time* to use their stories to accurately and appropriately portray their lives, informing government and the public about Aboriginal and Torres Strait Islander strengths and needs, cultures, and diversity.

Implicit within the participatory research paradigm is a strength-based research focus, contrasting the dominant deficit discourse permeating health research which focuses on disparities and serves to problematize Indigenous peoples [7, 63–65]. This strength-based approach has been strongly advocated for by community members and organisations, researchers, and increasingly by government [58, 59, 66–71]. *Footprints in Time* is purposefully designed to have a strength-based focus, as demonstrated by the study’s guiding research question; the focus on positive assets and resources of individuals, families, and communities; and the collection of information about culture [28]. Aligned with this strength-based research focus, most participant responses to why they participated in the study focused on strength – such as measuring children’s strength and progress, and their future as well as maintaining Aboriginal and Torres Strait Islander culture and improving policy. Another component of strength-based approaches is understanding and valuing diversity [28, 59, 72], to enable an accurate portrayal of the diverse lives and experiences of Aboriginal and Torres Strait Islander peoples. Multiple participants in our study commented on the importance of the study capturing diversity. *Footprints in Time* is currently the only national study of Aboriginal and Torres Strait Islander children [27]; the diverse sample in this study – in terms of life circumstances, experiences, location, and cultures – is critical to enabling a more accurate portrayal of Aboriginal and Torres Strait Islander life [17]. We note that the *Footprints in Time* study is not intended to be representative of all Aboriginal and Torres Strait Islander families, but rather to provide a snapshot of life in a diverse range of environments [27].

The building of connection and trust in research needs to be earned and developed over time; it is ‘difficult to establish, but easy to destroy’ ([62] p. 373). If trust is lost, participants are unlikely to continue to participate in research and share their stories [62]. *Footprints in Time* was developed and is conducted in partnership with Aboriginal and Torres Strait Islander communities and organisations [18, 27, 66]; this has been key to cultivation of participants’ and communities’ trust [5, 62]. An extensive community consultation process was undertaken during the study development phase, and outcomes of these consultations are reflected in the study design. For example, these consultations have ensured that the study focuses on Aboriginal and Torres Strait Islander community priorities; that it takes a holistic approach, considering the child’s wellbeing in the context of their family, community, and culture; that the study is conducted in a participatory and culturally respectful manner (including employment of Aboriginal and/or Torres Strait Islander interviewers, adherence to community protocols); and that it reflects the diversity of the population [22, 66, 73, 74]. The design of *Footprints in Time* inherently privileges and values Aboriginal and Torres Strait Islander voices and perspectives, which have so often been omitted from research [21, 75, 76]. Consistent with findings from Guillemin et al. [5], participants appreciated the opportunity to contribute their story to research and statistics, and to record aspects of their lives that might be lost if left unrecorded. Our findings indicate that this partnership approach and the study’s processes have supported maintenance of trust, and therefore study participation, over many years. Participants’ reflections on their reasons for engaging in the study suggest a sense of reciprocity and trust between families and the study team. Enjoyment in and perceived benefits to participation, including building relationships with the RAOs, were described as a key part of this.

Our findings support, and add strength to, previous literature on research participation and ethical research with Indigenous populations. This study is the first to incorporate perspectives from a large number (*n* = 160) of Aboriginal and Torres Strait Islander families and shows that Aboriginal and Torres Strait Islander people want to be involved in research when it is done ‘the right way’. While the current analysis focuses on the reasons participants contribute to the *Footprints in Time* study, there were a number of responses that emphasised the importance of future use of the data provided by participants. In particular, several caregivers indicated that it was important to them that the collected data were used, and used appropriately. Participatory methodologies are intended to not only engage participants in the research development and collection phases, but also in the analysis and implementation phases. Data collection for the *Footprints in Time* study remains ongoing, and analysis is underway. The next challenge for the study will be to engage in strategies that increase the translation of findings into policy and practice, in a way that is inclusive and relevant to Aboriginal and Torres Strait Islander peoples. 

### Strengths and limitations

Our study was limited to caregivers of Aboriginal and Torres Strait Islander children who decided to participate in *Footprints in Time*, and who participated in the 6th annual survey. We therefore lack perspectives (i.e. reasons for non-participation) from persons who chose not to continue to participate in the study, or who joined the study but did not participate in the 6th survey. Further, this research is based on analysis of the primary caregiver’s reasons for participating only; it does not incorporate the study child’s views, or the views of secondary or other caregivers.

Caregiver responses may have been influenced by social desirability bias, particularly as they were collected face-to-face with the RAOs. However, caregivers did have the option to indicate that they did not know why they stayed in the study, or to decline to response; this option was chosen by less than 10% of participants.

This analysis capitalised on free-text collected in a primarily quantitative survey, supporting previous literature on the potential value of these types of data [77, 78]. A random subset of responses were analysed and we stopped analysing data when saturation was reached, according to our protocol. There is the possibility that responses from participants in our random sub-sample may not be fully representative of all responses from the total sample. However, our sub-sample was selected at random and was generally similar to the overall Wave 6 sample in terms of demographic characteristics. The present analysis provides information based on a Aboriginal and Torres Strait Islander sample substantially larger and more diverse than previously published (*n* = 8) [5].

## Conclusion

Our findings support the assertion that Aboriginal and Torres Strait Islander people want to be involved in research when certain conditions are met. The *Footprints in Time* study has demonstrated the ability to recruit and retain a substantial number of families of Aboriginal and Torres Strait Islander children over six waves of data collection, from 2008 to 2013. This is enabled by the development and maintenance of a sense of connection and trust, and a mutually beneficial relationship between participants and the study. Specific processes and elements of participatory research can be implemented to cultivate reciprocity and trust in research, including building relationships between participants and researchers, involving local people in the design and data collection, ensuring individual and community benefit from the research, and ensuring that the research is of value to participants. Our findings reinforce the importance of doing research ‘the right way’, encompassing existing principles for the conduct of ethical research with Indigenous populations. Given historical power relations between Indigenous peoples and settler societies, meaningful investment in reciprocity at all research stages can help redress this negative past at the community level [61, 79]. Learnings from this study may be transferable to research with other Indigenous populations, to facilitate participation and retention in research, and support the generation of meaningful and relevant research findings that can contribute to improved wellbeing.

## Abbreviation

RAOs: Research Administration Officers
